# Antiretroviral Treatment Switch Among Treatment-Experienced People with HIV

**DOI:** 10.36469/001c.156180

**Published:** 2026-02-24

**Authors:** Benjamin Chastek, Uche Mordi, Mary J. Christoph, Lisa B. Le, Travis Lim, Sunil Majethia, Cassidy Trom, Joshua Cohen

**Affiliations:** 1 Optum, Eden Prairie, Minnesota; 2 Gilead Sciences, Inc., Foster City, California; 3 Independent Healthcare Analyst

**Keywords:** HIV, treatment switch, antiretroviral therapy, bictegravir, Medicare Advantage

## Abstract

**Background:**

HIV requires lifelong continuous antiretroviral therapy (ART) to prevent morbidity and mortality and to reduce onward transmission. People with HIV (PWH) may switch ART regimens for various clinical and nonclinical reasons. Identifying patterns of ART switching can inform shared decision-making and optimize regimen selection among treatment-experienced PWH.

**Objectives:**

To describe and compare treatment switching among PWH by regimen in a large US claims database, including a subset of PWH enrolled in Medicare Advantage.

**Methods:**

A retrospective analysis of closed US medical and pharmacy claims data was conducted in treatment-experienced PWH with commercial insurance or Medicare Advantage with Part D coverage from the Optum Research Database. PWH who switched to an ART regimen of interest (bictegravir/emtricitabine/tenofovir alafenamide [B/F/TAF], dolutegravir/lamivudine, dolutegravir/abacavir/lamivudine, dolutegravir + emtricitabine/tenofovir alafenamide, dolutegravir + emtricitabine/tenofovir disoproxil fumarate, and cabotegravir + rilpivirine) between July 1, 2017, and November 30, 2023, were included. Baseline characteristics were balanced across regimens in the overall study population using inverse probability treatment weighting. Time to switch/add-on was estimated using Kaplan-Meier analyses. Adjusted hazard ratios for the entire follow-up period were calculated using multivariable Cox proportional hazards models. Outcomes were assessed in the overall population and in the subset enrolled in Medicare Advantage.

**Results:**

Overall, 14 826 treatment-experienced PWH were included in the study. The proportion of PWH without switch/add-on after the first 12 months of follow-up was greater for those who were taking B/F/TAF compared with those taking the other regimens of interest (all *P* < .001). The risk of switch/add-on was significantly lower for those who were taking B/F/TAF vs those taking the other regimens of interest (all *P* ≤ .001). Findings were similar for the subset of Medicare Advantage enrollees.

**Discussion:**

Due to more effective treatments, PWH have increased life expectancy, and a growing proportion are qualifying for Medicare coverage. This analysis showed that PWH taking B/F/TAF, both in the overall population and the Medicare Advantage subset, maintained their initial treatment regimen longer and had a reduced risk of treatment switch/add-on compared with the other regimens of interest.

**Conclusions:**

These findings suggest that B/F/TAF has a lower likelihood of treatment switch/add-on for treatment-experienced PWH than other current ART regimens.

## INTRODUCTION

HIV is a lifelong condition requiring continuous therapy to prevent morbidity and mortality and to reduce onward transmission through virologic suppression.^1–5^ Integrase strand transfer inhibitor-based antiretroviral therapy (ART) regimens are the mainstay for initial therapy.^6^ People with HIV (PWH) may switch ART for various clinical (eg, virologic failure, suspected resistance, safety/tolerability) and nonclinical (eg, cost, stigma, access) reasons.^6,7^

Both persistence on and adherence to ART are critical for achieving and maintaining viral suppression^1,8^ and are of particular concern among older PWH who may have additional comorbidities.^9^ Over 50% of people diagnosed with HIV in the United States are aged 50 or more years, many of whom are Medicare-eligible based on age or long-term disability.^10,11^ Older PWH enrolled in Medicare Advantage, compared with those on commercial insurance, tend to have more chronic comorbidities and higher pill burdens, which are associated with suboptimal adherence.^12,13^

Identifying patterns of ART switching is important to help inform shared decision-making and optimize regimen selection among treatment-experienced PWH. This study aimed to describe and compare treatment switching among treatment-experienced PWH by regimen in a large US claims database and, in particular, a subset of PWH enrolled in Medicare Advantage.

## METHODS

### Study Design

This was a retrospective analysis of closed US medical and pharmacy claims data for treatment-experienced PWH with commercial insurance or Medicare Advantage with Part D coverage (Medicare Advantage) from the Optum Research Database. The index date was the earliest claim for an ART regimen of interest (index regimen) between July 1, 2017, and November 30, 2023 (identification period). The lookback period for HIV treatment experience began as early as January 1, 2007, which allowed for more than 10 years of data to determine ART history. The baseline period was defined as the 6 months before the index date. PWH were followed for at least 3 months from the index date until the end of the regimen or February 29, 2024, whichever came first. The regimens of interest were bictegravir (B)/emtricitabine (F)/tenofovir alafenamide (TAF), dolutegravir (DTG)/lamivudine (3TC), DTG/abacavir (ABC)/3TC, DTG + F/TAF, DTG + F/tenofovir disoproxil fumarate (TDF), and cabotegravir (CAB) + rilpivirine (RPV).

Institutional review board or independent ethics committee review and informed consent were not required for this secondary analysis of existing data.

### Study Population

Eligible study participants were aged 18 years or older; were not missing age, sex, geographic region, or insurance type data; had at least 1 claim with an HIV diagnosis during the identification period; had at least 1 claim for a regimen of interest during the identification period; and had continuous enrollment in a commercial or Medicare Advantage health plan with both pharmacy and medical benefits for at least 6 months prior to and for at least 3 months following the index date. To limit the study population to treatment-experienced PWH, only individuals who had evidence of ART use since January 1, 2007, with either (1) the most recent regimen being the same as the index regimen and a treatment gap of at least 90 days before starting the index regimen, or (2) the pre-index regimen being different from the index regimen, were selected.

### Variables

Study variables included baseline demographic and clinical characteristics. The main outcome was treatment switch/add-on during the follow-up period. Switch was defined as either (1) discontinuing some, but not all, components of the index regimen (with or without beginning new antiretroviral agent[s]), or (2) discontinuing all medications of the index regimen and starting a new regimen within 90 days of discontinuation. Add-on was defined as continuing all medications from the index regimen while adding at least 1 new medication; medications from the index regimen were carried forward to the second regimen if the individual had a refill of the medication after starting the second regimen but before experiencing a 90-day gap. The most common regimens at baseline (pre-index) and post-switch/add-on (post-index) were recorded.

### Statistical Analysis

Baseline demographic and clinical characteristics were recorded and analyzed descriptively. Inverse probability treatment weighting was conducted prior to switch/add-on analyses to balance baseline demographics, clinical measures, and healthcare costs and utilization across regimens in the overall study population.^14–16^ Kaplan-Meier analyses were used to estimate time to switch/add-on during the first 12 months of follow-up, and log-rank *P* values were used to assess differences in time-to-event distribution between groups at 12 months.^14–16^ Adjusted hazard ratios and corresponding 95% confidence intervals for the entire follow-up period were calculated using multivariable Cox proportional hazards models.^14,16^ *P* values <.05 were considered statistically significant. PWH were censored when they disenrolled from the health plan, reached the end of available data, or discontinued all index regimen components for at least 90 days. These analyses were conducted in the overall population and in the subset of PWH enrolled in Medicare Advantage.

## RESULTS

### Study Population

Among 113 705 individuals with an HIV diagnosis, 14 826 switched or restarted ART during the study period and met all inclusion criteria. Baseline demographic and clinical characteristics for the overall study population and the Medicare Advantage group have been previously described.^17^ Briefly, the mean (standard deviation [SD]) age of the overall study population was 52.7 (13.0) years, the majority of PWH were male (11 916; 80.4%), 6577 (44.4%) were White, and the mean (SD) Charlson Comorbidity Index score was 4.17 (2.20; **Table S1**). A total of 6131 (41.4%) PWH were enrolled in Medicare Advantage. Baseline characteristics were generally similar between the overall study population and the Medicare Advantage group, except the Medicare Advantage group had a higher age (mean [SD]: 61.3 [10.4] years) and Charlson Comorbidity Index score (mean [SD]: 4.92 [2.35]), and a lower proportion was male (70.1%).

In the overall study population, the most common baseline (pre-index) regimen was the same as the index regimen for the DTG/ABC/3TC (44.2%), DTG + F/TAF (18.7%), and DTG + F/TDF (24.9%) groups (**Table S2**). In the B/F/TAF, DTG/3TC, and CAB + RPV groups in the overall study population, the most common baseline regimens were elvitegravir/cobicistat/F/TAF (25.6%), DTG/ABC/3TC (29.0%), and B/F/TAF (19.5%), respectively.

### Treatment Switch/Add-on

**Figure 1.**
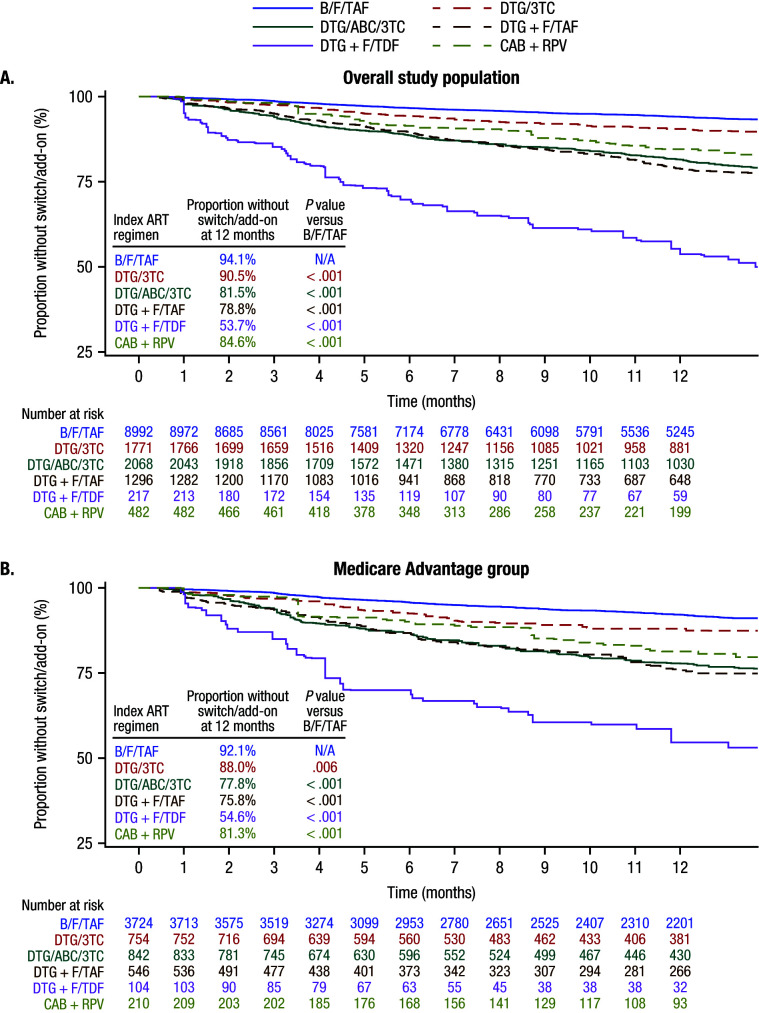
Time to Switch/Add-on^a^ for (**A**) Overall Study Population and (**B**) Medicare Advantage Group Abbreviations: 3TC, lamivudine; ABC, abacavir; ART, antiretroviral therapy; B, bictegravir; CAB, cabotegravir; DTG, dolutegravir; F, emtricitabine; Medicare Advantage, Medicare Advantage with Part D coverage; RPV, rilpivirine; TAF, tenofovir alafenamide; TDF, tenofovir disoproxil fumarate. ^a^Inverse probability treatment weighting was conducted prior to switch/add-on analyses to balance baseline demographics, clinical measures, and healthcare costs and utilization across regimens in the overall study population. Kaplan-Meier analyses were used to estimate time to switch/add-on during the first 12 months of follow-up, and log-rank *P* values were used to assess differences in time-to-event distribution between B/F/TAF and comparator regimens at 12 months.

At 12 months, in the overall study population, the proportion of PWH without treatment switch/add-on was significantly higher with B/F/TAF compared with DTG/3TC, DTG/ABC/3TC, DTG + F/TAF, DTG + F/TDF, and CAB + RPV (all *P* < .001; **Figure 1A**). Similarly, among the Medicare Advantage group, there was a significantly greater proportion of PWH without treatment switch/add-on with B/F/TAF compared with all other regimens (all *P* < .01; **Figure 1B**).

**Figure 2.**
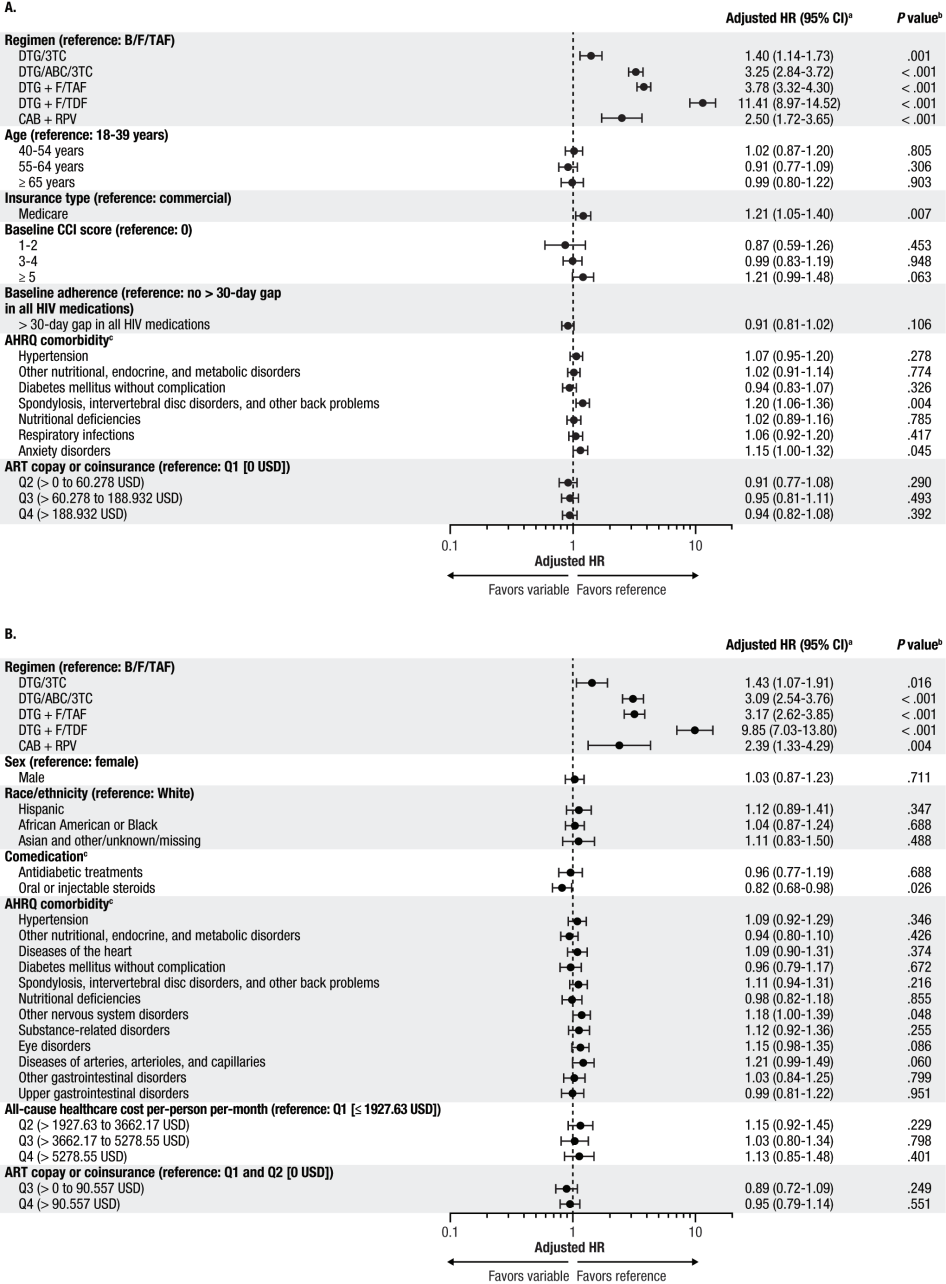
Risk of Switch/Add-on for (**A**) Overall Study Population and (**B**) Medicare Advantage Group Abbreviations: 3TC, lamivudine; ABC, abacavir; AHRQ, Agency for Healthcare Research and Quality; ART, antiretroviral therapy, B, bictegravir; CAB, cabotegravir; CCI, Charlson Comorbidity Index; CI, confidence interval; DTG, dolutegravir; F, emtricitabine; HR, hazard ratio; Medicare Advantage, Medicare Advantage with Part D coverage; PDC, proportion of days covered; RPV, rilpivirine; TAF, tenofovir alafenamide; TDF, tenofovir disoproxil fumarate. ^a^Inverse probability treatment weighting was conducted prior to switch/add-on analyses to balance baseline demographics, clinical measures, and healthcare costs and utilization across regimens in the overall study population. For the overall study population, HRs were adjusted by baseline demographic characteristics, CCI score, AHRQ comorbidities, AIDS-defining conditions, comedications, ambulatory counts, inpatient counts, and ART copay or coinsurance. For the Medicare Advantage group, HRs were adjusted by baseline demographic characteristics, CCI score, AHRQ comorbidities, AIDS-defining conditions, comedications, PDC, all-cause healthcare costs and utilization, and ART copay or coinsurance. AHRQ comorbidities were identified using the AHRQ Clinical Classification Software for *International Classification of Diseases, Ninth Revision, Clinical Modification/International Classification of Diseases, Tenth Revision, Clinical Modification* (hcup-us.ahrq.gov/toolssoftware/ccs/ccs.jsp). Adjusted HRs and corresponding 95% CIs for the entire follow-up period were calculated using multivariable Cox proportional hazards models. ^b^*P* values were calculated to determine statistically significant differences from the B/F/TAF group. ^c^For each comedication or AHRQ comorbidity, the reference was not having the respective comedication or AHRQ comorbidity.

In the overall study population, the risk of switch/add-on during the entire follow-up period was significantly higher with DTG/3TC, DTG/ABC/3TC, DTG + F/TAF, DTG + F/TDF, and CAB + RPV compared with B/F/TAF (all *P* ≤ .001; **Figure 2A**). The risk of switch/add-on was also higher among PWH in the overall population who had Medicare Advantage insurance compared with those who were commercially insured, as well as those with spondylosis, intervertebral disc disorders, and other back problems and those with anxiety disorders (all *P* < .05).

In the Medicare Advantage group, the risk of switch/add-on was significantly higher with DTG/3TC, DTG/ABC/3TC, DTG + F/TAF, DTG + F/TDF, and CAB + RPV compared with B/F/TAF (all *P* < .05; **Figure 2B**). Additionally, the risk of switch/add-on was lower among PWH in the Medicare Advantage group who were taking oral or injectable steroids and higher among those who had nervous system disorders (*P* < .05).

Among all PWH who switched/added-on to their regimen, B/F/TAF was the most common subsequent (post-index) regimen that PWH indexed on all other regimens switched to, with the proportion switching to B/F/TAF ranging from 25.4% for DTG + F/TDF to 42.8% for DTG + F/TAF (**Table S3**). Among those indexed on B/F/TAF who later switched, the most common subsequent regimen was DTG/3TC (22.7%).

## DISCUSSION

In this retrospective claims analysis of treatment-experienced PWH with commercial or Medicare Advantage insurance, PWH who received B/F/TAF experienced a longer period before treatment switch/add-on and had a lower risk of switch/add-on than PWH who received DTG/3TC, DTG/ABC/3TC, DTG + F/TAF, DTG + F/TDF, or CAB + RPV. Similar findings were observed in the subset of PWH enrolled in Medicare Advantage.

Over the past two decades, Medicare Advantage enrollment has markedly increased, with 54% (32.8 million people) of the eligible Medicare population enrolled in Medicare Advantage by 2024.^18^ Given the increased life expectancy of PWH, due to more effective treatments, a growing proportion are qualifying for Medicare based on age, with PWH accounting for 23% of Medicare enrollees in 2020.^11^ While some unique circumstances of Medicare enrollees have been associated with suboptimal adherence and treatment gaps,^19^ the present study found that treatment switch/add-on patterns by ART regimen in the Medicare Advantage group were generally similar to those observed in the overall study population, with B/F/TAF being associated with the lowest switch/add-on rate.

Descriptive analysis of the most common ART regimens at baseline (pre-index) showed that many PWH switched from older to newer integrase strand transfer inhibitor-based regimens, which have higher barriers to resistance, better tolerability, and fewer drug-drug interactions.^20^ In addition, there were PWH who switched from multi-tablet to single-tablet regimens, which have a lower pill burden and are associated with better treatment persistence and adherence.^21,22^ Notably, B/F/TAF was the most common regimen that PWH switched to, even among those who were indexed on recently approved regimens.

The current study has limitations. First, the reasons for both index regimen selection and switch/add-on were not observable in the database; additional studies are required to better understand treatment characteristics associated with switch/add-on. Second, the noninformative censoring assumption for the Cox model was not specifically tested. Third, the Medicare Advantage population in this study may not be representative of the broader Medicare population with HIV, many of whom are covered by Medicare Fee-for-Service or are dually eligible for Medicare and Medicaid.^11^ Lastly, formulary inclusion and detailed insurance coverage information were not available in the database to allow for the inference of insurer-driven reasons for switch/add-on by ART regimen.

Understanding real-world ART switching patterns with contemporary regimens can assist in optimizing treatment selection in different populations and consequently help to achieve and maintain viral suppression. Findings from this study suggest that B/F/TAF has a lower likelihood of treatment switch/add-on for treatment-experienced PWH compared with other ART regimens, and it is a common therapy to switch to for those previously on DTG-based regimens or CAB + RPV.

### Disclosures

B.C. and L.B.L. are employees of Optum and stockholders of UnitedHealth Group. U.M., M.J.C., T.L., S.M., and C.T. are employees and stockholders of Gilead Sciences, Inc. J.C. is an independent healthcare analyst who was formerly employed by Tufts University.

## Supplementary Material

Online Supplementary Material

## Data Availability

The Optum Research Database was commercially licensed from the data vendor. The data contained in the Optum Research Database include proprietary elements owned by Optum and, therefore, cannot be broadly disclosed or made publicly available at this time. The disclosure of these data to third-party clients assumes certain data security and privacy protocols are in place and that the third-party client has executed the standard license agreement, which includes restrictive covenants governing the use of the data.
