# Supplementary material for: Antiretroviral Treatment Switch Among Treatment-Experienced People with HIV
Source: J Health Econ Outcomes Res. 2026 Feb 24;13(1):73–8. doi: 10.36469/001c.156180 (PMC12940528; doi:10.36469/001c.156180)
Supplement: Online Supplementary Material [file jheor_2026_13_1_156180_332679.pdf]

Journal of Health Economics and Outcomes Research

Online Supplementary Material

Antiretroviral Treatment Switch Among Treatment-Experienced People with HIV. *JHEOR*. 2026;13(1):73-78. doi:10.36469/jheor.2026.156180

Table S1: Baseline Demographic and Clinical Characteristics in the Overall Study Population of Treatment-Experienced PWH (Unweighted)

Table S2: Ten Most Common Baseline ART Regimens by Index ART Regimen in the Overall Study Population of Treatment-Experienced PWH (Pre-Index)

Table S3: Most Common Post-Switch/Add-On ART Regimens by Index Regimen in the Overall Study Population of Treatment-Experienced PWH (Post-Index)

This supplementary material has been provided by the authors to give readers additional information about their work.

This is an open-access article distributed under the terms of the Creative Commons Attribution 4.0 International License (CCBY-4.0). View this license's legal deed at http://creativecommons.org/licenses/by/4.0 and legal code at http://creativecommons.org/licenses/by/4.0/legalcode for more information.

Table S1. Baseline Demographic and Clinical Characteristics in the Overall Study Population of Treatment-Experienced PWH (Unweighted)

Characteristic	Overall (N = 14,826)	B/F/TAF (n = 8992)	DTG/3TC (n = 1771)	DTG/ABC/3TC (n = 2068)	DTG + F/TAF (n = 1296)	DTG + F/TDF (n = 217)	CAB + RPV (n = 482)
Age, y, mean (SD)	52.7 (13.0)	52.7 (12.9)	54.1 (13.2)	51.9 (13.5)	53.1 (12.6)	49.4 (14.0)	52.8 (12.9)
Male, n (%)	11,916 (80.4)	7322 (81.4)	1404 (79.3)	1631 (78.9)	1021 (78.8)	161 (74.2)	377 (78.2)
Race/ethnicity,[a] n (%)							
White	6577 (44.4)	4021 (44.7)	794 (44.8)	868 (42.0)	592 (45.7)	97 (44.7)	205 (42.5)
Hispanic	2056 (13.9)	1274 (14.2)	276 (15.6)	258 (12.5)	163 (12.6)	24 (11.1)	61 (12.7)
African American or Black	4720 (31.8)	2815 (31.3)	472 (26.7)	791 (38.2)	419 (32.3)	66 (30.4)	157 (32.6)
Asian	265 (1.8)	147 (1.6)	43 (2.4)	38 (1.8)	28 (2.2)	4 (1.8)	5 (1.0)
Insurance type, n (%)							
Commercial	8695 (58.6)	5284 (58.8)	1027 (58.0)	1269 (61.4)	764 (59.0)	137 (63.1)	214 (44.4)
Medicare Advantage	6131 (41.4)	3708 (41.2)	744 (42.0)	799 (38.6)	532 (41.0)	80 (36.9)	268 (55.6)
CCI score, mean (SD)	4.17 (2.20)	4.15 (2.20)	4.10 (2.11)	4.01 (2.25)	4.53 (2.29)	3.97 (2.47)	4.47 (1.81)

Abbreviations: 3TC, lamivudine; ABC, abacavir; B, bictegravir; CAB, cabotegravir; CCI, Charlson Comorbidity Index; DTG, dolutegravir; F, emtricitabine; Medicare Advantage, Medicare Advantage with Part D coverage; PWH, people with HIV; RPV, rilpivirine; SD, standard deviation; TAF, tenofovir alafenamide; TDF, tenofovir disoproxil fumarate.

[a]In total, 267 PWH (152 receiving B/F/TAF, 41 receiving DTG/3TC, 31 receiving DTG/ABC/3TC, 30 receiving DTG + F/TAF, 6 receiving DTG + F/TDF, and 7 receiving CAB + RPV) had a race/ethnicity of "other/unknown." Additionally, 941 PWH (583 receiving B/F/TAF, 145 receiving DTG/3TC, 82 receiving DTG/ABC/3TC, 64 receiving DTG + F/TAF, 20 receiving DTG + F/TDF, and 47 receiving CAB + RPV) had no available data on race/ethnicity.

Table S2. Ten Most Common Baseline ART Regimens by Index ART Regimen in the Overall Study Population of Treatment-Experienced PWH (Pre-Index)[a]

Index ART Regimen											
**B/F/TAF (n = 8992)**		**DTG/3TC (n = 1771)**		**DTG/ABC/3TC (n = 2068)**		**DTG+F/TAF (n = 1296)**		**DTG+F/TDF (n = 217)**		**CAB+RPV (n = 482)**	
**Baseline ART Regimen**	**n (%)**	**Baseline ART Regimen**	**n (%)**	**Baseline ART Regimen**	**n (%)**	**Baseline ART Regimen**	**n (%)**	**Baseline ART Regimen**	**n (%)**	**Baseline ART Regimen**	**n (%)**
EVG/COBI/F/TAF	2298 (25.6)	**DTG/ABC/3TC**	**513 (29.0)**	**DTG/ABC/3TC**	**915 (44.2)**	**DTG + F/TAF**	**242 (18.7)**	**DTG + F/TDF**	**54 (24.9)**	**B/F/TAF**	**94 (19.5)**
EFV/F/TDF	1080 (12.0)	EVG/COBI/F/TAF	262 (14.8)	EFV/F/TDF	275 (13.3)	**DTG + F/TDF**	**176 (13.6)**	DTG	33 (15.2)	EVG/COBI/F/TAF	53 (11.0)
**B/F/TAF**	**1079 (12.0)**	**B/F/TAF**	**190 (10.7)**	EVG/COBI/F/TAF	82 (4.0)	DTG	165 (12.7)	RAL + F/TDF	18 (8.3)	**DTG/ABC/3TC**	**39 (8.1)**
**DTG/ABC/3TC**	**582 (6.5)**	RPV/F/TAF	106 (6.0)	EVG/COBI/F/TDF	40 (1.9)	EFV/F/TDF	100 (7.7)	EFV/F/TDF	16 (7.4)	CAB+RPV + B/F/TAF	38 (7.9)
RPV/F/TAF	430 (4.8)	**DTG/3TC**	**90 (5.1)**	ATV + ABC/3TC	34 (1.6)	EVG/COBI/F/TAF	73 (5.6)	EVG/COBI/F/TAF	13 (6.0)	DTG/RPV	35 (7.3)
**DTG + F/TAF**	**414 (4.6)**	EFV/F/TDF	71 (4.0)	ABC/3TC + DTG	31 (1.5)	**DTG/ABC/3TC**	**48 (3.7)**	**DTG + F/TAF**	**9 (4.1)**	RPV/F/TAF	34 (7.1)
EVG/COBI/F/TDF	303 (3.4)	**DTG + F/TAF**	**66 (3.7)**	RPV/F/TAF	30 (1.5)	RPV/F/TAF	34 (2.6)	EVG/COBI/F/TDF	8 (3.7)	DRV/COBI/F/TAF	16 (3.3)
RPV/F/TDF	154 (1.7)	DTG/RPV	46 (2.6)	RAL + ABC/3TC	26 (1.3)	**B/F/TAF**	**30 (2.3)**	**B/F/TAF**	**5 (2.3)**	**DTG/3TC**	**16 (3.3)**
DRV/COBI + F/TAF	143 (1.6)	EVG/COBI/F/TDF	27 (1.5)	EFV + ABC/3TC	25 (1.2)	ATV + F/TAF	23 (1.8)	**DTG/ABC/3TC**	**5 (2.3)**	**DTG + F/TAF**	**11 (2.3)**
RAL + F/TAF	142 (1.6)	DTG/ABC/3TC + DTG/3TC	24 (1.4)	DTG/ABC/3TC + DRV/COBI	23 (1.1)	RAL + F/TAF	23 (1.8)	ATV + DTG + F/TDF	4 (1.8)	CAB+RPV + EVG/COBI/F/TAF	9 (1.9)

Abbreviations: 3TC, lamivudine; ABC, abacavir; ART, antiretroviral therapy; ATV, atazanavir; B, bictegravir; CAB, cabotegravir; COBI, cobicistat; DRV, darunavir; DTG, dolutegravir; EFV, efavirenz; EVG, elvitegravir; F, emtricitabine; PWH, people with HIV; RAL, raltegravir; RPV, rilpivirine; TAF, tenofovir alafenamide; TDF, tenofovir disoproxil fumarate.
[a]Bolded type denotes regimens of interest. Shading denotes the baseline regimen is the same as the index regimen. Medications in individual claims are separated by a + symbol, except in the case of CAB+RPV (which is based on a single claim).

Table S3. Most Common[a] Post-Switch/Add-On ART Regimens by Index Regimen in the Overall Study Population of Treatment-Experienced PWH (Post-Index)[b]

**Index ART Regimen**											
**B/F/TAF (n = 801)**		**DTG/3TC (n = 167)**		**DTG/ABC/3TC (n = 445)**		**DTG+F/TAF (n = 404)**		**DTG+F/TDF (n = 114)**		**CAB+RPV (n = 78)**	
**Subsequent ART Regimen**	**n (%)**	**Subsequent ART Regimen**	**n (%)**	**Subsequent ART Regimen**	**n (%)**	**Subsequent ART Regimen**	**n (%)**	**Subsequent ART Regimen**	**n (%)**	**Subsequent ART Regimen**	**n (%)**
**DTG/3TC**	**182 (22.7)**	**B/F/TAF**	**46 (27.5)**	**B/F/TAF**	**142 (31.9)**	**B/F/TAF**	**173 (42.8)**	**B/F/TAF**	**29 (25.4)**	**B/F/TAF**	**20 (25.6)**
**CAB+RPV**	**65 (8.1)**	**CAB+RPV**	**13 (7.8)**	**DTG/3TC**	**93 (20.9)**	DTG[d,e]	58 (41.4)	**DTG + F/TAF**	**26 (22.8)**	**DTG/3TC**	**9 (11.5)**
DTG/RPV	45 (5.6)	DTG/3TC + CAB + RPV[c]	10 (6.0)	–	–	**DTG/3TC**	**30 (7.4)**	DTG[d,f]	22 (19.3)	CAB + RPV + B/F/TAF[c]	7 (9.0)
**DTG + F/TAF**	**41 (5.1)**	–	–	–	–	–	–	–	–	EVG/COBI/F/TAF	5 (6.4)
EVG/COBI/F/TDF	40 (5.0)	–	–	–	–	–	–	–	–	DTG/RPV	4 (5.1)

Abbreviations: 3TC, lamivudine; ABC, abacavir; ART, antiretroviral therapy; B, bictegravir; CAB, cabotegravir; COBI, cobicistat; DTG, dolutegravir; EVG, elvitegravir; F, emtricitabine; PWH, people with HIV; RPV, rilpivirine; TAF, tenofovir alafenamide; TDF, tenofovir disoproxil fumarate.

[a]Subsequent ART regimens received by ≥5.0% of PWH in each index regimen group were reported.

[b]Bolded type denotes regimens of interest. Medications in individual claims are separated by a + symbol, except in the case of CAB+RPV (which is based on a single claim).

[c]CAB+RPV may have been billed prior to administration, resulting in CAB+RPV and another regimen appearing together in the same cell.

[d]Subsequent ART regimens with only DTG were identified as monotherapies without a nucleoside reverse transcriptase inhibitor backbone.

[e]Among those who switched from DTG+F/TAF to DTG monotherapy, 30 PWH restarted DTG+F/TAF and 10 switched to a new complete regimen (most commonly DTG+F/TDF [n = 4]) within 90 days of stopping DTG monotherapy. Eighteen PWH either did not have a subsequent complete regimen or the next complete regimen identified was initiated >90 days after stopping DTG monotherapy.

[f]Among those who switched from DTG+F/TDF to DTG monotherapy, 4 PWH restarted DTG+F/TDF and 8 switched to DTG+F/TAF within 90 days of stopping DTG monotherapy. Five PWH either did not have a subsequent complete regimen or the next complete regimen identified was initiated >90 days after stopping DTG monotherapy. Five PWH switched to a nonstandard regimen within 90 days of stopping DTG monotherapy.
